# Changing trends in penile prosthesis implantation in China and an overview of postoperative outcomes from a single center

**DOI:** 10.1186/s12610-024-00228-z

**Published:** 2024-08-09

**Authors:** Chenwang Zhang, Haowei Bai, Chenkun Shi, Huirong Chen, Peng Li, Yuhua Huang, Huixing Chen, Fujun Zhao, Chencheng Yao, Zheng Li, Erlei Zhi

**Affiliations:** 1 State Key Laboratory of Reproductive Medicine and Offspring Health, Nanjing Medical University, Nanjing, 211166 China; 2 Department of Andrology, the Center for Men’s Health, Urologic Medical Center, Shanghai General Hospital, Shanghai Jiao Tong University School of Medicine, Shanghai, 200080 China

**Keywords:** Erectile Dysfunction(ED), Penile prosthesis, Complication, Satisfaction, Dysfonction érectile (DE), Prothèse pénienne, Complications, Satisfaction

## Abstract

**Background:**

Surgical penile prosthesis implantation (PPI) procedures have only recently been introduced to mainland China, with the overall number of such procedures having been conducted to date remaining relatively low. Accordingly, relatively little remains known with respect to the annual trends in PPI. Accordingly, this study was developed with the goal of clarifying these trends across different hospitals in mainland China, while also providing a single-center overview of post-PPI patient outcomes.

**Results:**

To identify males in mainland China who had undergone PPI, a retrospective review of data from January 2019 – October 2023 was conducted. This approach revealed an increase in the total PPI caseload from 120 in 2019 to 413 within the first 10 months of 2023. Over this same interval, the number of surgeons performing PPI rose from 33 to 74. A retrospective review of the 112 patients who had undergone PPI at Shanghai General Hospital from 2019–2023 revealed that these patients had a median age of 39 [27–63] years, and PPI treatment led to a significant increase in median International Index of Erectile Function-5 (IIEF-5) scores from a baseline value of 10.23 ± 1.26 to a post-treatment value of 22.6 ± 2.73. The underlying causes of erectile dysfunction for these patients included vasculogenic factors (58/112; 51.8%), diabetes mellitus (21/112; 18.8%), and injuries to the spinal cord or pelvis (14/112; 12.5%). The overall rates of satisfaction with the PPI reported by patients and their partners were 93.0% and 90.4%, respectively, and the 3-year PPI survival rate for this cohort was 87%.

**Conclusion:**

These data highlight a rising trend in the number of PPI being performed in China, with these steadily increasing rates since 2019 emphasizing the increasingly high levels of acceptance of this procedure by patients and clinicians as a means of treating erectile dysfunction. However, the expertise is restricted to a small number of surgeons. Even so, it is a safe and efficacious approach to managing severe erectile dysfunction for patients in China, and when performed by experienced surgeons based on standardized protocols, low complication rates can be achieved while providing patients and their sexual partners with high levels of satisfaction.

**Supplementary Information:**

The online version contains supplementary material available at 10.1186/s12610-024-00228-z.

## Résumé

**Contexte:**

Les procédures chirurgicales d’implantation de prothèses péniennes (IPP) n’ont été que récemment introduites en Chine continentale, le nombre total de procédures de ce type ayant été effectuées à ce jour restant relativement faible. On ne sait donc encore que relativement peu de choses sur les tendances annuelles de l’IPP. La présente étude a été développée dans le but de clarifier ces tendances dans différents hôpitaux de Chine continentale, tout en fournissant une vue d’ensemble des résultats des patients post-IPP dans un seul centre.

**Résultats:**

Afin d’identifier les hommes de Chine continentale qui avaient subi un IPP, une recherche rétrospective des données a été effectuée de janvier 2019 à octobre 2023. Cette approche a révélé une augmentation du nombre total de cas d’IPP de 120 en 2019 à 413 au cours des 10 premiers mois de 2023. Au cours de cette même période, le nombre de chirurgiens pratiquant des IPP est passé de 33 à 74. L’étude rétrospective des 112 patients qui avaient subi un IPP à l’hôpital général de Shanghai de 2019 à 2023 a révélé qu’ ils avaient un âge médian de 39 [27–63] ans, et que le traitement par IPP a entraîné une augmentation significative des scores médians de l’indice international de la fonction érectile-5, qui sont passés d’une valeur de base de 10,2 ± 1,3 à une valeur post-traitement de 22,6 ± 2,7. Les causes sous-jacentes de la dysfonction érectile chez ces patients comprenaient des facteurs vasculogéniques (58/112; 51,8%), un diabète (21/112; 18,8%) et des lésions de la moelle épinière ou du bassin (14/112; 12,5%). Les taux globaux de satisfaction à l’égard de l’IPP, rapportés par les patients et leurs partenaires, étaient respectivement de 93,0% et 90,4%, et le taux de survie à 3 ans de l’IPP dans cette cohorte était de 87%.

**Conclusion:**

Ces données mettent en évidence une tendance à la hausse du nombre d’IPP pratiquées en Chine; ces taux en constante augmentation depuis 2019 soulignent les niveaux de plus en plus élevés d’acceptation de cette procédure par les patients et les cliniciens comme moyen de traitement de la dysfonction érectile. Cependant, l’expertise est limitée à un petit nombre de chirurgiens. Malgré cela, il s’agit d’une approche sûre et efficace pour gérer la dysfonction érectile sévère pour les patients en Chine, et lorsqu’elle est effectuée par des chirurgiens expérimentés sur la base de protocoles standardisés, de faibles taux de complications peuvent être atteints tout en offrant aux patients et à leurs partenaires sexuels des niveaux élevés de satisfaction.

## Introduction

Over the past five decades, penile prosthesis implantation (PPI) has emerged as an effective means of treating severe erectile dysfunction (ED) in men [1, 2]. By the year 2025, ED is predicted to impact more than 322 million individuals owing to the aging of the global population and rising rates of obesity, pelvic trauma, injury to the spinal cord, Peyronie’s disease [3], diabetes, and psychogenic illnesses [4], driving higher rates of PPI [5–7].

PPI is a procedure that is performed more frequently in wealthier developed nations as compared to developing nations. In contrast to many other nations, both private insurers and the government provide coverage for PPI in the USA, where an estimated 25,000–30,000 of these procedures are performed each year [8]. Differences in rates of PPI treatment have been observed as a function of healthcare infrastructure, ethnicity, geographic region, insurance coverage, and socioeconomic conditions. China is the developing nation with the highest population, and is home to 4 and 21 times the number of people living in the USA and UK, respectively [9]. While it is an important procedure, trends related to the implementation of PPI treatment remain poorly understood in mainland China.

The Chinese State Food and Drug (CSFD) administration first provided approval for penile prostheses in 2000, but there has been little information published regarding PPI treatment among Chinese individuals to date [10]. Since 2015, the AMS700 CX and CXR prostheses from the Boston Scientific Corporation (BSC) have been available in mainland China. Given that domestically produced penile prostheses face high failure rates, these AMS700 implants have emerged as the near-exclusive choice for PPI treatment in mainland China. Statistical information regarding these PPI procedures only first became available in 2019. The present study provides an overview of the upward trends in PPI procedural volume in mainland China, revealing that these procedures have primarily been performed by a limited number of professionals in relatively wealthy cities since 2019. Shanghai General Hospital was also selected as a representative facility to provide insight regarding the current PPI procedural landscape and prognostic outcomes for treated patients.

## Patients and methods

### Study design

Data from BSC were used to conduct a retrospective analysis of the number of PPI procedures in mainland China conducted from January 2019 – October 2023, allowing for the identification of males in mainland China who had undergone this form of surgery. Additionally, the medical records of patients who underwent this PPI procedure at Shanghai General Hospital during this same interval were assessed. All patients provided written informed consent. Routine preoperative examinations for these patients included analyses of the international index of erectile function-5 score (IIEF-5), nocturnal penile tumescence and rigidity (NPTR), audiovisual sexual stimulation (AVSS), and serum levels of a range of hormones (Testosterone, estradiol, prolactin, luteinizing hormone, follicle-stimulating hormone) [10]. Patients from our center with severe ED who were unaffected by uncontrolled diabetes/hypertension, psychiatric illnesses, severe renal and/or hepatic dysfunction, or genital infections were included in this study. Severe ED included cases in which other medical management strategies had failed or were intolerable, and with the need to confirm that ED was organic in nature through Doppler ultrasonography, or cavernosograph [10].

### Data collection

GDP data and information related to numbers of hospitals, surgeons, and assistant practitioners were obtained from the China Statistical Yearbook published by the National Bureau of Statistics (https://www.stats.gov.cn/sj/ndsj/). Chinese PPI procedures were obtained through data from BSC. The Chinese GDP data were accessed through the International Monetary Fund (https://www.imf.org/en/Home).

### Surgical treatment

PPI procedures were performed via a standard approach under general anesthesia [11, 12]. Catheterization was used to empty the bladder, disinfecting the hands of the operating surgeon for > 15 min using a povidone-iodine scrub. A scrotal or penoscrotal incision was then used to implant an AMS 700 prosthesis, irrigating the corpus cavernosum with an antibiotic-containing saline solution before implantation. To reduce the risk of scrotal hematoma or infection after surgery, a vacuum drain was placed. Patients were admitted to the hospital for 5–7 days after surgery.

### Follow up

Patients were instructed regarding the operation of their prostheses, and were permitted to initiate sexual intercourse six weeks following PPI procedural completion. Patients also completed a follow-up questionnaire composed of three parts, including one using the IIEF-5 to assess ED status [10], the use of Bhojwani’s score to assess sexual satisfaction [13], and the use of the Clavien-Dindo system to evaluate complications [14].

### Statistical analyses

Data were analyzed with SPSS 13.0, and were compared using chi-square tests or rank-sum tests. *P* < 0.05 was the threshold for significance.

## Results

### PPI surgery rates are on the rise in Mainland China

**Fig. 1:**
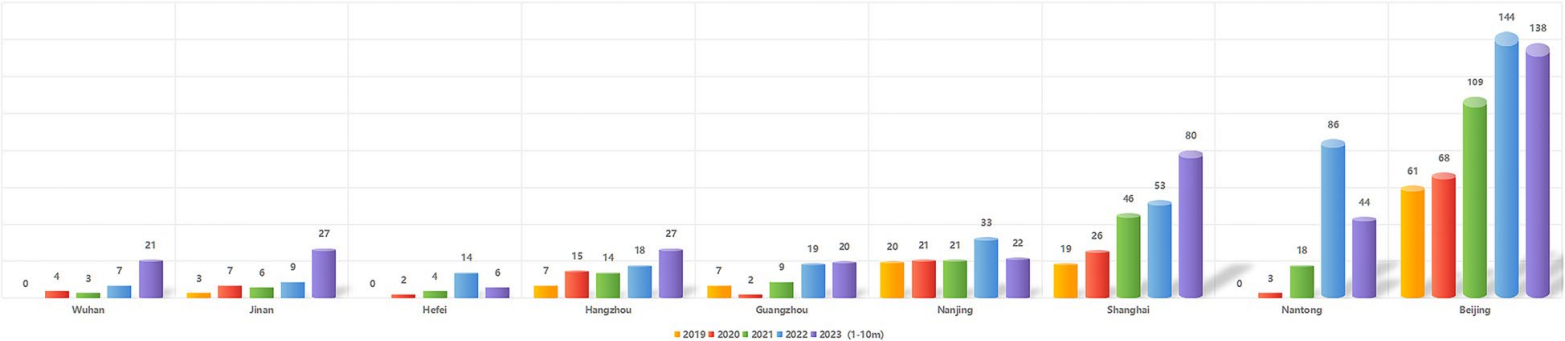
The annual PPI surgery caseloads for 9 cities with more than 10 PPI surgical cases in the first 10 months of 2023. Nine total cities were identified in which more than 10 PPI surgical procedures were performed during the first 10 months of 2023, including Beijing, Nantong, Shanghai, Nanjing, Guangzhou, Hangzhou, Hefei, Jinan, and Wuhan. PPI: penile prosthesis implantation

Between the beginning of 2019 and October of 2023, the number of PPI procedures has risen steadily in mainland China, even though the latter three of these years overlapped with the COVID-19 pandemic. Cities in which more than 10 PPI procedures had been performed during the first 10 months of 2023 included Beijing, Nantong, Shanghai, Nanjing, Guangzhou, Hangzhou, Hefei, Jinan, and Wuhan. The largest number of procedures was performed in Beijing, with 138 procedures in 2023 as of October (Fig. 1).

### PPI is concentrated in a few large cities and a minority of surgeons in Mainland China

#### The relationship between PPI and city GDP values

**Fig. 2:**
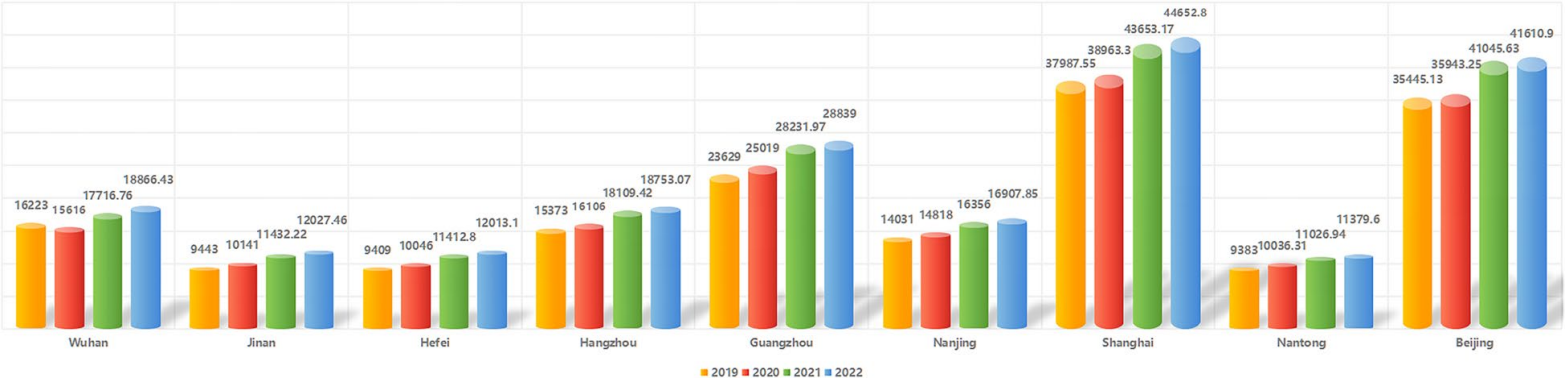
The GDPs of the 9 cities with high PPI caseloads from 2019–2022. The annual GDP values for each of these four years were assessed in 9 cities in which more than 10 PPI surgical procedures were performed during the first 10 months of 2023. GDP: Gross Domestic Product. Units in the figure are shown in: *10^8^￥

PPI data revealed that procedural volume was positively correlated with the GDP values of these different Chinese cities. This is unsurprising, given that each procedure can cost $20,000 such that both patients and clinicians must take economic factors into consideration. Of the 9 cities in which more than 10 PPI procedures had been performed through October of 2023 mentioned above, 6 were among the cities with the 10 highest GDP values in 2022 (Fig. 2), while the remaining 3 were among the cities with the 25 highest GDP values. Notably, Jinan and Jefei are the respective capitals of Shandong and Anhui provinces. Nantong stands as something of an exception among these cities, with all PPI procedures in this city having been conducted by a single surgeon skilled in this procedure.

#### PPI Expertise is restricted to a limited number of practitioners in Mainland China

**Fig. 3:**
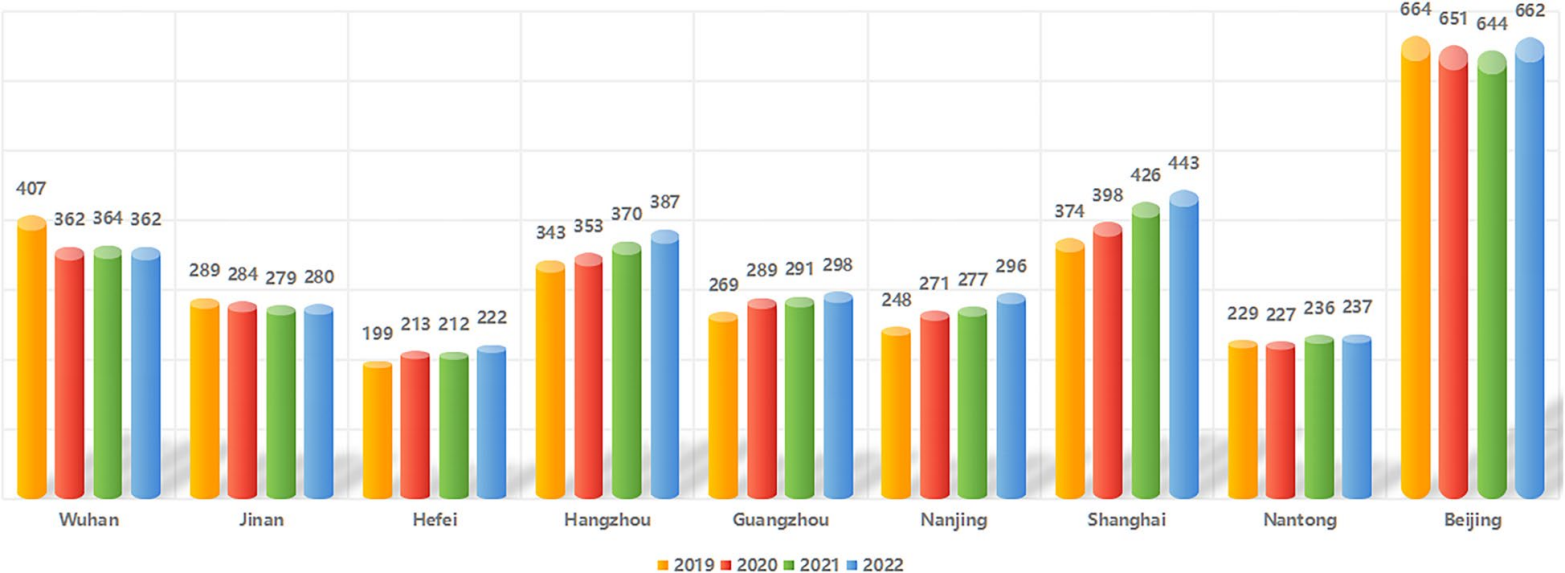
The numbers of hospitals in the 9 cities with high PPI caseloads from 2019–2022. Numbers of hospitals across the indicated 9 cities in which more than 10 PPI surgical procedures were performed during the first 10 months of 2023

**Fig. 4:**
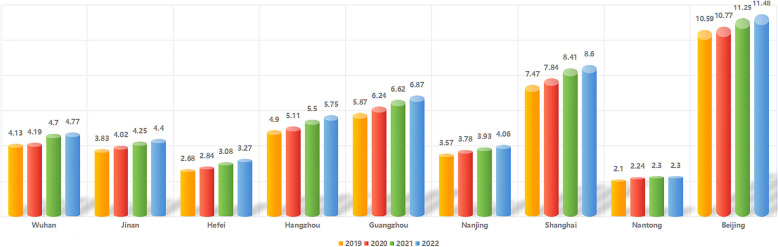
Numbers of practitioners and assistant practitioners in cities with high PPI caseloads from 2019–2022. Numbers of practitioners during each of these four years were assessed in 9 cities in which more than 10 PPI surgical procedures were performed during the first 10 months of 2023. Numbers in the figure are shown in the following units: *10.^4^

PPI is one of the most complex andrological surgeries, but there has been a marked increase in the number of practitioners performing this procedure in mainland China from 2019–2023, rising from 33 to 74 surgeons. However, this experience remains extremely restricted given that even within the top 9 cities where the largest numbers of PPI procedures have been performed to date, there are more than 100 hospitals and over 10,000 physicians and assistant practitioners (Figs. 3 and 4). However, there are fewer than 100 surgeons proficient in the PPI procedure, underscoring the relative exclusivity of mastery over this procedure, which has only been cultivated among a limited subset of surgeons.

### Surgical outcomes of PPIs in our center

#### Demographics

Shanghai has one of the three highest PPI caseloads in mainland China, and Shanghai General Hospital is the leading hospital where these procedures are performed. In total, 112 patients have undergone PPI procedures at our center to date, with a median age of 39[27–63] years. Prior to treatment, the median IIEF-5 score for these 112 patients was 10.23 ± 1.26, with 81 (72.3%) having previously undergone PDE5i. The underlying causes of severe ED for these patients included vasculogenic factors (58/112; 51.8%), diabetes mellitus (21/112; 18.8%), and injuries to the spinal cord or pelvis (14/112; 12.5%). All patients underwent telephone-based follow-up with a median duration of 36 months.

The average lengths of the left and right cavernosa for these patients were 16.0 ± 5.38 cm and 16.1 ± 4.85 cm, respectively. Of these cases 68 (60.7%) were classified as instances of primary ED that had been present since initial attempts at sexual intercourse, whereas 44 were secondary to injuries or other diseases. Of the males included in this study, 5 with ED that was secondary to pelvic fractures presented with urethral stricture and underwent simultaneous PPI. For one patient exhibiting extensive corporal fibrotic scarring as a result of ischemic priapism, this scar tissue was resected, after which corporal reconstruction was performed (Table 1).

**Table 1:** Baseline characteristics for patients from our hospital who underwent PPI

Characteristic	Study group (*N* = 112)
Age(years) [range]	39 [27–63]
IIEF-5 scores (Mean ± SD)	10.23 ± 1.26
Course of ED before PPI (months)[range]	32 [17–46]
Primary etiology (%)	
Vasculogenic	51.8% (58/112)
Diabetes mellitus	18.8% (21/112)
Pelvic trauma /Spinal cord injury	12.5% (14/112)
Iatrogenic	0.9% (1/112)
Peyronie’s disease	6.25% (7/112)
Ischaemic priapism	0.90% (1/112)
Psychogenic	2.60% (3/112)
Unknown	6.25% (7/112)
Previous ED treatment, n (%)	
PDE5-I	72.3% (81/112)
Vacuum	10.7% (12/112)
Traditional herb medicine	46.4% (52/112)
Unknown	4.46% (5/112)
Follow-up (months) [range]	36 [1–60]

*IIEF-5* international index of erectile function-5 scores, *SD* standard deviation, *ED* erectile dysfunction, *PPI* penile prosthesis implantation, *PDE5-I* phos-phodiesterase type 5 inhibitor

#### IIEF-5 scores

Within the 6-month postoperative period, all 112 patients underwent IIEF-5 score evaluations in the clinic. All patients reported being able to engage in regular sexual intercourse with their partners, and they exhibited significant improvements in IIEF-5 scores from preoperative values of 10.23 ± 1.26 to postoperative values of 22.6 ± 2.73 (*P* < 0.05) (Table 2).

**Table 2:** PPI outcomes for patients from our hospital

Characteristic	Study group (*N* = 112)	
IIEF-5 scores ( Mean ± SD)	22.6 ± 2.23	
Satisfaction score	Patients (*n* = 89, %)	Sexual partners (*n* = 62,%)
10	17.8% (16/89)	16.1% (10/62)
9	13.5% (12/89)	12.9% (8/62)
8	18.0% (16/89)	19.4% (12/62)
7	23.5% (21/89)	25.8% (16/62)
6	20.2% (18/89)	16.2% (10/62)
5	2.2% (2/89)	4.8% (3/62)
4	3.7% ( 3/89)	3.2% (2/62)
1–3	1.1% (1/89)	1.6% (1/62)
Complication, n (%)	13.4%(15/112)	
Grade I	0.9% (1/112)	
Grade II	1.8% (2/112)	
Grade IIIb	10.7% (12/112)	
3-year PPI survival rate	87%	

*SD* standard deviation, *PPI* penile prosthesis implantation

#### Sexual satisfaction

Of the included subjects, 89 patients consented to analyses of sexual satisfaction, as did 62 of their sexual partners. These subjects reported significant increases in satisfaction, with slightly higher levels of satisfaction for patients relative to their partners although the difference was non-significant (*P* > 0.05). When satisfaction was analyzed with Bhojwani’s sexual satisfaction score, overall 93.0% and 90.4% satisfaction rates with the PPI procedure were respectively reported by patients and their partners (Table 2).

#### Complications

Of the patients in this cohort, 13.4% (15/112) experienced at least one complication. These included 0.9% (1/112) of patients with grade I complications as a result of urethral injury, 1.8% (2/112) with grade II complications as a result of hematomas, 10.7% (12/112) with grade IIIb complications owing to revisions and infections. The overall 3-year PPI survival rate for this cohort was 87% (Table 2) [1].

## Discussion

The number of PPI surgical procedures performed annually has risen steadily since 2019 in mainland China, when data first became available, suggesting that the COVID-19 pandemic did not hamper these treatment efforts. Notably, COVID-19 infections have been linked to erectile difficulties [15–17], contributing to higher ED prevalence among males. The pandemic also exposed greater psychological and physical stress on many people, both of which can contribute to the incidence of ED. These factors may have fueled greater interest in the surgical management of this condition. Indeed, the resilience of the upward trend in PPI case volume suggests that acceptance of this procedure is growing among both surgeons and patients in light of its efficacy. Even so, this procedure remains very expensive relative to the income of most individuals in mainland China, and current medical insurance guidelines do not provide reimbursement for PPI such that treated patients and physicians must take these economic factors into consideration. Economic factors are thus a key determinant of PPI adoption as a treatment strategy, consistent with the observed positive correlation between the number of PPI cases and the GDP values of the surveyed Chinese cities. Financial considerations are thus closely tied to the overall prevalence of this surgical procedure at present.

PPI is among the most complex surgeries performed in the andrology field [18]. While the rise in the number of surgeons performing PPI procedures from 33 to 74 during the analyzed period (2019–2023) suggests that expertise in this highly specialized surgery continues to grow, such proficiency remains highly concentrated among a relatively small number of surgeons. Indeed, the top 9 cities where the most PPI procedures have been performed to date are home to hundreds of hospitals and more than 10,000 physicians and assistants, underscoring the extremely small percentages of practitioners who are experienced in this procedure. Even if demand for PPI treatment is on the rise across medical facilities, this concentrated expertise among a small group of surgeons highlights potential challenges with respect to procedural accessibility such that targeted training initiatives may be essential to meet with rising levels of demand throughout mainland China.

To provide further perspective on the use of PPI and associated outcomes, we evaluated data from patients who had undergone PPI as a treatment for severe ED in our hospital. All 112 of these patients were able to engage in regular sexual intercourse with their partners after the PPI procedure, with significant improvements in IIEF-5 score values from 10.23 ± 1.26 to 22.6 ± 2.73. The median age of these patients was 39 years of age, in contrast to what has been reported for Western populations, and the primary causes of PPI were vasculogenic (51.8%), diabetes mellitus (18.8%), and spinal cord injuries or pelvic trauma (12.5%) in mainland China [19, 20]. While there is evidence suggesting that the application of PPI in mainland China remains limited owing to a lack of patient acceptance together with high costs, younger and middle-aged patients experiencing severe ED exhibit greater odds of procedural acceptance so that they can maintain sexual relationships, marriages, and familial stability.

Cavernosum length is associated with height and ethnicity, with the average lengths of cavernosa among Asian males potentially being less than the average for males from Western nations (19–22 cm) [21]. Indeed, the average left and right cavernosum lengths for this study cohort were 16.0 ± 5.38 cm and 16.1 ± 4.85 cm, respectively, in line with the average of 16–17 cm that has been reported for Korean populations [10]. This emphasizes the need to produce prostheses with smaller cylinders better suited to the anatomy of Asian males, as current prostheses are manufactured using data from Western populations.

PPI treatment is aimed at improving quality of life by allowing males to resume engaging in sexual activity. A retrospective analysis performed by Varvalheira et al. found that male satisfaction was related to sexual function after PPI treatment, with factors that were significantly associated with satisfaction including unnatural sensations, a lack of partner satisfaction, a decrease in penile length, and retarded ejaculation [22, 23]. The patients and partners included in the present study presented with high levels of satisfaction (93% and 90.4%, respectively), in line with what has been reported previously [24]. Satisfaction rates rose between the initial visit and the final follow-up. These findings underscore the need for training in the use of implanted prostheses for patients and their sexual partners. As with any implant, complications including infections, malfunctions, and damage to peripheral organs can occur following PPI procedures [25]. Of the PPI patients from our hospital, 13.4% experienced at least one complication, including 0.9%, 1.8%, and 10.7% of patients having experienced grade I, II, and IIIb complications as a result of urethral injury, hematomas, and revisions/infections, respectively. None of these patients experienced severe bowel, bladder, or vascular injuries, although one patient who experienced intraoperative urethral injury had to undergo urethral catheterization. The most serious postoperative complication was infection, with prostheses becoming infected in 4.5% of cases, in line with past reports [26].

### Limitations

This study was retrospective in design. As PPI surgeries have only recently begun being conducted in mainland China, the numbers of these procedures remain limited both at the national level and in our hospital. Moreover, mainland China lacks any interconnected medical record management system at the national level, hampering access to detailed follow-up records for all patients who have undergone PPI procedures. As such, our team is actively contacting the 10 leading hospitals in mainland China with respect to PPI surgical caseloads in order to initiate a multicenter clinical study aimed at gathering more detailed follow-up information from individuals in mainland China who have undergone penile prosthesis procedures.

## Conclusion

In summary, PPI procedures are steadily growing more common in mainland China. Such procedures are a safe and effective means of managing severe ED in Chinese patient populations, and when implantation is performed by an experienced surgeon based on standardized PPI protocols, this can lower the risk of complications while improving postprocedural sexual satisfaction for both patients and their partners.

### Supplementary Information

Supplementary Material 1.

## Data Availability

The datasets used and analyzed during the current study are available from the corresponding author on reasonable request.

## Abbreviations

ED: Erectile dysfunction
IIEF-5: International index of erectile function-5 scores
PPI: Penile prosthesis implantation
BSC: Boston scientific corporation
